# Primary immunosuppressive TNI-based conditioning regimens in pediatric patients treated with haploidentical hematopoietic cell transplantation

**DOI:** 10.1007/s00066-021-01840-y

**Published:** 2021-09-02

**Authors:** D. Wegener, P. Lang, F. Paulsen, N. Weidner, D. Zips, M. Ebinger, U. Holzer, M. Döring, F. Heinzelmann

**Affiliations:** 1 Department of Radiation Oncology, University Clinic of Tuebingen, Tuebingen, Germany; 2 Department of Paediatrics I, Hematology and Oncology, University Clinic of Tuebingen, Tuebingen, Germany; 3 Department of Radiation Oncology, Clinic of Esslingen, Esslingen, Germany

**Keywords:** Primary conditioning, Total nodal irradiation, Engraftment, Toxicity, Raditherapy in pediatric patients

## Abstract

**Purpose:**

This retrospective analysis aims to address the toxicity and efficacy of a modified total nodal irradiation (TNI)-based conditioning regimen before haploidentical hematopoietic cell transplantation (HCT) in pediatric patients.

**Materials and methods:**

Patient data including long-term follow-up were evaluated of 7 pediatric patients with malignant (*n* = 2) and non-malignant diseases (*n* = 5) who were treated by a primary TNI-based conditioning regimen. TNI was performed using anterior/posterior opposing fields. All patients received 7 Gy single-dose TNI combined with systemic agents followed by an infusion of peripheral blood stem cells (*n* = 7). All children had haploidentical family donors.

**Results:**

Engraftment was reached in 6/7 children after a median time of 9.5 days; 1 child had primary graft failure but was successfully reconditioned shortly thereafter. After an average follow-up time of 103.5 months (range 8.8–138.5 months), event-free (EFS) and overall survival (OS) rates were 71.4% and 85.7%, respectively. One child with a non-malignant disease died 8.8 months after transplantation due to a relapse and a multiple organ failure. Follow-up data was available for 5/6 long-term survivors with a median follow-up (FU) of 106.2 months (range 54.5–138.5 months). Hypothyroidism and deficiency of sexual hormones was present in 3/5 patients each. Mean forced expiratory volume in 1 s (FEV1) after TNI was 71%; mean vital capacity (VC) was 78%. Growth failure (< 10th percentile) occurred in 2/5 patients (height) and 1/5 patient (weight). No secondary malignancies were reported.

**Conclusion:**

In this group of patients, a primary single-dose 7 Gy TNI-based conditioning regimen before HCT in pediatric patients allowed sustained engraftment combined with a tolerable toxicity profile leading to long-term OS/EFS. Late toxicity after a median FU of over 9 years includes growth failure, manageable hormonal deficiencies, and acceptable decrease in lung function.

## Introduction

Haploidentical hematopoietic cell transplantation (HCT) is a potentially curative treatment for several life-threatening malignant and benign hematological diseases [1]. The effect of non-myeloablative modified total nodal irradiation (TNI) to achieve immunosuppression and the feasibility and favorable outcome of TNI-based (re-)conditioning regimes have been described previously for both malignant and non-malignant diseases [2–4]. However, long-term outcome and toxicity data are scarce in this high-risk population [5, 6] and a variety of TNI regimes are in use [7, 8]

This retrospective analysis aims to address the efficacy and toxicity of a 7 Gy single-dose TNI-based conditioning regimen before HCT on pediatric patients with various life-threatening diseases.

## Materials and methods

An analysis of 7 pediatric patients treated by a 7-Gy single-dose TNI conditioning regimen before primary HCT for various benign or malignant diseases (Tables 1 and 2) was performed. All patients were treated at the University Hospital of Tuebingen between 2006 and 2011. Regular follow-up (FU) examinations at the children’s hospital were performed yearly over up to 10 years and these records were analyzed. The modified TNI technique consists of a supradiaphragmatic mantle field including the thymus and an infradiaphragmatic “inverted-y” field encompassing the spleen while shielding non-lymphoid tissues in the head, chest (e.g., lung), abdomen (e.g., kidneys and liver), and pelvis. This method was described in detail in a previous publication 2. All patients received TNI combined with systemic agents (Tables 1 and 2) followed by an infusion of peripheral blood stem cells (*n* = 7). All patients had haploidentical family donors. Grafts were depleted of T and B cells (cluster of differentiation 3 and 19 [CD3/CD19] depletion) in all pediatric patients with malignant and non-malignant diseases to mitigate (or reduce) the risk of graft vs. host disease (GVHD). Various GVHD prophylaxes were employed. Long-term immunosuppressive therapy had been administered in 5/7 patients before transplantation as a prior therapy. Neutrophil engraftment was defined as the first of three consecutive days of which the absolute neutrophil count was > 0.5 *10^9^/l. Toxicity was evaluated from posttreatment check-ups. Cardiac toxicity was assessed from pathological results in ECG or cardiac echo examinations. Thyroid toxicity, kidney toxicity, and hormonal status were defined by pathological blood parameters (TSH, fT3, fT4; creatinine/glomerular filtration rate; IGF‑1, IGFBP‑3; testosterone, LH, FSH). Lung toxicity was estimated by forced expiratory volume in 1 s (FEV1) and vital capacity (VC) at last examination. Growth and height are given in age- and gender-corrected percentiles.

**Table 1:** Patient characteristics

Disease			
*Malignant*	Myelodysplastic syndrome		*n* =2
*Benign*	Alpha-thalassemia		*n* =1
	B‑cell deficiency		*n* =1
	Autoimmunogenic neutropenia		*n* =1
	Aplastic anemia		*n* =2
*Immunosuppressive therapy prior to transplantation*	–		*n* =5
*Age at transplantation*	Median 8.33 years (range 4.58–14.33 years)		
*TNI regime*	1 × 7.0 Gy		*n* =7
*Therapeutic agents used for reconditioning:*	Fludarabine, melphalan, thiotepa, muromonab-CD3		*n* =5
	Fludarabine, melphalan, thiotepa, anti-thymocyte globulin		*n* =2
*Cells transplanted per kg body weight (median, range)*	Mononuclear cells in × 10^8^	13.77 (8.12–25.73)	*n* =7
	CD34+ progenitor cells in × 10^6^	17.34 (6.06–28.22)	*n* =7
	CD3+ cells in × 10^4^	667.4 (3.47–2953.46)	*n* =7
*Engraftment*	6/7 patients		
*Time from TNI to engraftment*	Median 9.5 days (range 9–10 days)		

*TNI* total nodal irradiation, *CD* cluster of differentiation

**Table 2:** Treatment characteristics by patient

Disease	Sex	Systemic agents for conditioning (additional to 7 Gy TNI)	Dosage	Long-term therapy
Myelodysplastic syndrome	f	Fludarabine	5 × 40 mg/m^2^	Rituximab
		Melphalan	1 × 140 mg/m^2^	Mycophenolate mofetil
		Thiotepa	1 × 10 mg/kg	Prednisolone
		Muromonab	1 × 0.01 mg/kg	
		Anti-thymocyte globulin	2 × 2 mg/kg	
Severe combined immunodeficiency with B cell deficiency and hypogammaglobulinemia and alterations in the T cell spectrum	f	Fludarabine	5 × 40 mg/m^2^	Mycophenolate mofetil
		Melphalan	1 × 140 mg/m^2^	
		Thiotepa	1 × 10 mg/kg	
		Muromonab	1 × 0.01 mg/kg	
		Anti-thymocyte globulin	2 × 2 mg/kg	
Alpha-thalassemia	f	Fludarabine	4 × 40 mg/m^2^	Mycophenolate mofetil
		Melphalan	1 × 70 mg/m^2^	Prednisolone
		Thiotepa	1 × 10 mg/kg	
		Muromonab	8 × 0.1 mg/kg	
Autoimmunogenic neutropenia	f	Fludarabine	4 × 40 mg/m^2^	Rituximab
		Melphalan	1 × 70 mg/m^2^	
		Thiotepa	1 × 10 mg/kg	
		Muromonab	8 × 0.1 mg/kg	
Aplastic anemia	f	Fludarabine	4 × 40 mg/m^2^	Mycophenolate mofetil
		Melphalan	2 × 70 mg/m^2^	Prednisolone
		Thiotepa	1 × 10 mg/kg	
		Muromonab	26 × 0.1 mg/kg	
		Prednisolone	8 × 4 mg/kg	
Aplastic anemia	f	Fludarabine	4 × 40 mg/m^2^	Ciclosporin A
		Melphalan	2 × 70 mg/m^2^	Mycophenolate mofetil
		Thiotepa	1 × 10 mg/kg	Prednisolone
		Anti-thymocyte globulin	3 × 5 mg/kg	
Myelodysplastic syndrome	m	Fludarabine	4 × 40 mg/m^2^	Mycophenolate mofetil
		Melphalan	2 × 70 mg /m^2^	
		Thiotepa	1 × 10 m/kg	
		Muromonab	24 × 0.1 mg/kg	
		Prednisolone	7 × 4 mg/kg	

*TNI* total nodal irradiation*, f* female, *m* male

### Statistical analysis

Survival data for overall survival (OS), event-free survival (EFS), and non-relapse mortality (NRM) were calculated according to the Kaplan–Meier product limit method. Kaplan–Meier analyses were performed using Medcalc® (version 18.5, MedCalc Software, Ostend, Belgium). OS was defined as the time from HCT to death from any cause. EFS was defined as the time interval from the date of HCT to the date of relapse/progression of the underlying disease or death from any cause. NRM was defined as death from any cause without prior relapse or progression.

## Results

### Engraftment

After a median follow-up time of 9.5 days, 6/7 children engrafted (range 9–10 days). One child had graft failure but successful re-transplantation 37 days after the first TNI including a 2 × 2 Gy total body re-irradiation and was alive at last FU (LFU). Patient characteristics are given in Tables 1 and 2.

### Survival outcome

**Fig. 1:**
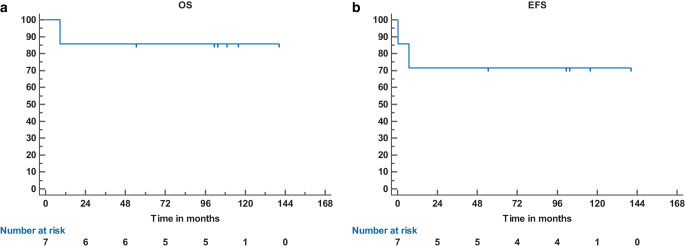
Kaplan–Meier curves of the patient cohort. X‑axis: time in months. Y‑axis: survival probability. Number at risk given with each curve. **a** Overall survival (*OS*) of the cohort. **b** Event-free survival (*EFS*) of the cohort

The average FU time was 103.5 months (range 8.8–138.5 months). At LFU, event-free (EFS) and overall survival (OS) rates were 71.4% (standard error 17.1%) and 85.7% (standard error 13.2%), respectively. Kaplan–Meier graphs are shown in Fig. 1. Rates for 1‑, 3‑, and 5‑year OS were 85.7% each. NRM did not occur in this cohort. Despite initial sustained engraftment, one child with a complex combined immunodeficiency with B cell deficiency (Table 1) died 8.8 months after transplantation due to a relapse and (re-)treatment-related septic multiple organ failure.

### Acute and long-term toxicity

All patients had grade 1–2 mucositis and fever and several manageable infections occurred. Severe acute toxicity (grade≥ 3 CTCAE [9]) was reported in three children and consisted of mucositis G3 (*n* = 2) and general dermatitis G4 (*n* = 1). In all cases, the patients recovered after cortisone treatment and phototherapy (dermatitis G4). No acute lung toxicity, no clinical signs of veno-occlusive disease, and no cardiac or neurotoxicity were detected.

FU data were available for 5/6 long-term survivors with a median FU of 106.2 months (range 54.5–138.5 months). One patient with aplastic anemia returned to her country of origin after the transplantation and could not be assessed for the evaluation of long-term toxicity. Results are shown in Tables 3 and 4. Hypothyroidism and insufficiency of sexual hormones was present in 3/5 patients each. Mean FEV1 after TNI was 71%, mean VC was 78%. No cardiac toxicity or chronic kidney failure occurred. Growth failure (< 10th percentile) was present in 2/5 patients (height) and 1/5 patient (weight). When compared to the growth and weight percentiles of each patient at the time of TNI, a mean decrease of −43 and −39 percentile points was found for growth and weight, respectively (Table 4). No secondary malignancies were reported in the surviving fraction with a median follow-up of 9.1 years (range 8.6–10.2 years).

**Table 3:** Late toxicity after 7‑Gy TNI-based conditioning

Item		No. of patients with available FU data	Median FU (months, range)	Pathological result (*n*)
Alive at LFU	6/7	5	106.2 (54.5–138.5)	1 patient with B cell deficiency died
Cardiac toxicity	ECG	5	108.9 (103.5–122.3)	0
	Cardiac echo	5		0
Thyroid toxicity		5	108.9 (103.5–122.3)	3 (hypothyroidism, oral substitution)
Renal toxicity		5	108.9 (103.5–122.3)	0
Hormonal status (other than thyroid)		5	108.9 (103.5–122.3)	3 (insufficiency of sexual hormones)
*Mean result (range)*				
Lung toxicity	FEV1 (%)	5	108.9 (103.5–122.3)	71.3% (66.3–77%)
	VC (%)	5		77.8% (68–88%)

*TNI* total nodal irradiation, *FU* follow up, *LFU* last FU, *ECG* electrocardiography, *FEV1* forced expiratory volume in 1 s, *VC* vital capacity

**Table 4:** Further late toxicity after 7‑Gy TNI-based conditioning at LFU

Toxicity item	Number of patients with available data	Results	
Age-appropriate growth	5	Weight > P50	*n* = 2
		Weight < P50	*n* = 2
		Weight < P25	*n* = 1
		Weight < P3	*n* = 0
		Height > P50	*n* = 0
		Height < P50	*n* = 1
		Height < P25	*n* = 3
		Height < P3	*n* = 1
Mean change in growth percentile from TNI to LFU^a^	5	Height −43 percentiles (range −89 to +4) Weight −39 percentiles (range −54 to −3)	–
Orthopedic toxicity	5	Slipped capital femoral epiphysis	*n* = 1
Hypertonia	5	–	*n* = 2
Neurologic/psychiatric toxicity	5	Depression	*n* = 1
Other toxicity reported at LFU	5	Bilateral hearing impairment	*n* = 1
Secondary malignancies	6	0	

*TNI* total nodal irradiation, *LFU* last follow up, *P* percentile, *n* number of patients

^a^Percentiles calculated with age- and gender correction

## Discussion

### Engraftment and survival outcome

In this retrospective analysis of 7 pediatric patients undergoing single-dose 7 Gy TNI-based primary conditioning before allogeneic HCT, 6/7 children achieved sustained engraftment leading to long-term survival in this mixed cohort as evidenced by the stable OS and EFS plateaus reached after transplantation (OS 85.7% at LFU, median FU 9.1 years). Due to the lack of an identical sibling or unrelated donor, haploidentical HCT was performed, which is nowadays considered a standard of care conditioning regimen [10]. It is currently difficult to compare our long-term outcome results with previous published data due to small-sized series, heterogeneity of patients and underlying diseases, and different FU times. In adult patients with severe aplastic anemia treated similarly with 7.5-Gy single-dose TNI and a conditioning regime containing ATG, Park et al. could demonstrate excellent engraftment (88/90 patients) and survival rates (OS 100%) after a median FU of 49.6 months [11]. Ocanto et al. report similar engraftment rates and low acute toxicity in an approach using fractionated total lymphatic irradiation in 25 pediatric patients with various malignant hematological diseases but did not report long-term follow-up [7].

### Toxicity

Immunosuppressive approaches before transplantation such as the regimens in our study have been developed in order to enable crucial engraftment while reducing organ toxicity [2, 12–14], which is associated with the use of myeloablative conditioning regimens [15, 16]. Of interest is the comparison of toxicity of TNI to regimens containing total body irradiation (TBI). Late effects of TBI can include interstitial pneumonitis in up to 30% of patients which can limit OS [17], veno-occlusive liver disease, cataracts in up to 50% of patients [18], hypothyroidism [19], chronic kidney insufficiency [20], and an increased risk for secondary malignancies [7, 15, 17].

We are able to provide acute and late toxicity analyses with a median FU of 9.1 years of surviving patients. One patient died 8.8 months after HCT due to a relapse and the toxicity related to the systemic re-treatment. There is no evidence of a death related to the toxicity of a TNI procedure. Heart, kidney, and lung function parameters at LFU were at acceptable rates, not requiring treatment. Outcome and toxicity rates are in accordance with another cohort study of 33 retransplanted pediatric patients (malignant diseases *n* = 25, benign *n* = 8) having undergone 7‑Gy TNI for reconditioning after graft failure [4] in our clinic. In both studies, growth failure was a frequent and arguably the most relevant late toxicity, with a mean difference of −43 and −39 percentile points for growth and weight, respectively, between TNI and LFU. The high rates of growth failure compared to the literature of pediatric patients undergoing HCT is worrying [21] and might be related to the higher biologically effective 7‑Gy single-dose treatment compared to fractionated regimes (2-Gy equivalent dose assuming an α/β of 4.5 Gy: 12.4 Gy) [7, 22]. Yet this dose is still at the lower end of the spectrum which is known to cause stunting of vertebral growth and well within the recent recommendations of Hoeben et al. [23]. To differentiate between the influence of systemic therapies or TNI on growth, an analysis of length of bones within the TNI field (i.e., vertebrae) compared to lengths of extremities, which were not irradiated, might proof helpful. Of note, in the detailed examinations of the children’s hospital, no visual discrepancy of this kind was mentioned for any patient. Additionally, the percentage of patients with hormonal insufficiencies hints towards a strong influence of growth hormone insufficiency on total growth. Yet no bone scans were available. Therefore, especially younger children might benefit from fractionated TNI regimens [23, 24]. In addition, modern radiation techniques (e.g., tomotherapy, IMRT) and/or fractionated TNI may lead to even lower rates of late toxicities [24, 25].

### Secondary malignancies

Radiation therapy in children is justifiably considered to bear a high risk of secondary malignancies, especially in organs that are very sensitive to radiation exposure such as the thyroid, salivary gland, mammary gland, bone, and connective tissue [26, 27]. We focused particularly on this issue. Noteworthily, in this long-term study with a median follow-up of 9.1 years (range 4.5 to 11.5 years) and regularly performed detailed examinations, no secondary malignancies have yet been reported. However, since especially solid secondary malignancies are known to have latencies of several decades (e.g., breast cancer), lifelong clinical follow-up is recommended [28].

### Limitations

Patient data were analyzed retrospectively and the study population is small and quite heterogeneous; therefore, outcome and toxicity data for a given entity cannot be predicted seriously. Further prospective, multicenter studies are desirable to identify the most promising conditioning regimen comparing single-dose versus fractionated TNI with chemotherapy and antibody-derived protocols, also considering the toxicity profile.

## Conclusion

In this group of pediatric patients, single-dose 7‑Gy TNI-based conditioning before allogenic hematopoietic cell transplantation allowed sustained engraftment combined with a tolerable toxicity profile leading to long-term OS/EFS. Late toxicity after a median LFU of over 9 years includes growth failure, manageable hormonal deficiencies, and acceptable decrease of lung function. No secondary malignancies were reported.
